# Editorial: The Impact of School Food Consumption on Children’s Cognition, Educational Attainment and Social Development

**DOI:** 10.3389/fpubh.2017.00204

**Published:** 2017-08-17

**Authors:** Lesley Drake, Riccardo Russo, Margaret (Greta) A. Defeyter

**Affiliations:** ^1^Partnership for Child Development, Imperial College London, London, United Kingdom; ^2^University of Essex, Colchester, United Kingdom; ^3^Northumbria University, Newcastle upon Tyne, United Kingdom

**Keywords:** school feeding, cognition, education attainment, social development, food consumption, government investment

**Editorial on the Research Topic**

**The Impact of School Food Consumption on Children’s Cognition, Educational Attainment and Social Development**

As a result of national and global initiatives driven by the Millennium Development Goals, more kids go to primary school than ever before. Now, in the era of the Sustainable Development Goals, the aim is to get more children through secondary education. The title of our editorial says it all: healthy children learn better. For children to learn, they need to be in school, and for them to benefit the most from the education at hand, they need to be healthy. This elevates the importance of continuing good health in schools.

Health status affects cognitive ability, educational attainment, quality of life, and the ability to contribute to society. Some of the most common health conditions of childhood have consequences for education. Health and nutrition interventions can support vulnerable children throughout key stages of their development in middle childhood and adolescence (1, 2). Research has shown that the average IQ loss for children with these conditions can range from 3.7 IQ points per child with untreated worm infections to 6.0 IQ points for children with anemia. Together, these prevalent conditions are estimated to translate into the equivalent of between 200 million and 500 million years of school lost due to ill health in LMICs each year (3).

Interventions for these common health conditions can have long-term economic benefits. Estimates show that poor students in areas where these conditions are prevalent would gain the equivalent of 0.5–2.5 extra years of schooling if their health benefited from appropriate interventions. Sustaining the benefits across multiple years of schooling could improve cognitive abilities by 0.25 SDs, on average; extrapolating the benefits of improved accumulation in human capital could translate to roughly a 5% increase in earning capacity over the life course.

School feeding programs are gaining increasing recognition for their twin roles as a long-term social protection investment as well as acting as a productive safety net for children and their families in the short term, and as an effective mechanism to reach the most vulnerable (4, 5). They are a social safety net, providing income support to families through the provision of food. Building on an existing and pervasive infrastructure can reduce start-up costs, accelerate program implementation, and reduce programmatic costs, while optimizing the benefits for education, increasing access to care for the most marginalized, and encouraging girls to attend and stay in school.

Most school feeding programs in sub-Saharan Africa operate around localized community-based supply chains. This is a “win–win” in that the parents of the children that we want back in school are invariably the smallholder farmers that also benefit from these programs, and the investment in rural economies contributes to national food security (6, 7).

It is estimated that more than 368 million schoolchildren are provided with school meals every day (8). The total investment in the intervention is projected to be as much as US$75 billion annually (8), largely from government budgets.

Analysis of school feeding funding has shown that national governments in low-income countries are increasing their investments in school feeding, with a rise of 12% in 4 years, from 6% in 2008 to 18% in 2012 (8). As the wealth of countries increases, the cost of school feeding compared to the cost of education decreases. This results in school feeding becoming relatively more affordable, with school feeding costing on average 68% of education costs in low-income countries, 24% in lower-middle-income countries, and 11% in upper-middle-income and high-income countries (8).

School feeding can serve to protect earlier investments in child welfare, buffering the effects of early shocks and contributing to the continuum of interventions from childhood through adolescence and into adulthood. Furthermore, school feeding also has the potential to address emerging issues such as the nutrition transition and could be integrated with other school health interventions for greater impact. Despite being one of the most expensive school health and nutrition interventions, school feeding is by far the most widely implemented by governments around the world. There is recognition of the multiple strong returns in investment: it provides a social safety net for poor children. School feeding works: children are learning, they are healthier, and they have the potential to become productive members of society with lifelong healthy dietary behaviors.

Schools are a cost-effective platform for providing simple, safe, and effective health interventions to school-age children and adolescents (9–11). The economies of scale, coupled with the efficiencies of using existing infrastructure, and the potential to administer additional interventions through this delivery mechanism have enormous potential. Schools can reach an unprecedented number of children and adolescents and play a key role in national development efforts by improving both child health and education. Because schools are at the heart of all communities, we have an opportunity to use the school as a sustainable, scalable option for simple health service delivery, and provision of services outside normal school hours (Harvey-Golding et al.; Sarr et al.).

This e-book provides examples from across the globe where school feeding and nutrition interventions are making a difference.

## Author Contributions

All three authors contributed to the editorial development of this e-book, and the concept behind the thinking of how nutritious school feeding impacts on children’s cognition, education attainment, and social development. All three authors have had continual engagement with all authors of the papers in the e-book.

## Conflict of Interest Statement

The authors declare that the research was conducted in the absence of any commercial or financial relationships that could be construed as a potential conflict of interest.
